# A double-blind, randomized trial on the effect of a broad-spectrum dietary supplement on key biomarkers of cellular aging including inflammation, oxidative stress, and DNA damage in healthy adults

**Published:** 2017-01-03

**Authors:** Lucas C. Lages, Johanna Lopez, Ana Maria Lopez-Medrano, Steven E. Atlas, Ana H. Martinez, Judi M. Woolger, Eduard Tiozzo, Janet Konefal, Armando J. Mendez, Herbert G. Simoes, John E. Lewis

**Affiliations:** 1 *Department of Psychiatry and Behavioral Sciences, University of Miami Miller School of Medicine, Miami, FL, United States*; 2 *Department of Medicine, University of Miami Miller School of Medicine, Miami, FL, United States*; 3 *Department of Family Medicine and Community Health, University of Miami Miller School of Medicine, Miami, FL, United States*

**Keywords:** dietary supplement, DNA damage, inflammation, oxidative stress, older adults, senescence

## Abstract

**Background and Aim:** Nutritional approaches that ameliorate cellular senescence may have the potential to counteract the effects of chronic disease. This study will investigate the effect of the Healthycell dietary supplement on markers of inflammation, oxidative stress, and DNA damage.

**Methods:** Thirty adults between the ages of 18 and 55 were enrolled and randomly assigned to one of the two study conditions (n = 15 Healthycell and n = 15 placebo). Subjects participated in a four-week intervention and were assessed at baseline, four weeks, and six weeks (after a two-week washout period).

**Results:** Pro-inflammatory cytokine interleukin (IL)-1α (t = 2.033; mean difference = −3.97 pg/ml; SE = 2.0; 95% CI: −8.0, −0.3; Cohen’s d = 0.77; p = 0.05) decreased, while soluble cytokine receptors sTNFR-I (t = 2.057; mean difference = 52.39 pg/mL; SE = 18.5; 95% CI: 5.2, 99.6; Cohen’s d = 0.53; *p* = 0.03) and sTNFR-II (t = 1.739; mean difference = 208.71 pg/ml; SE = 72.0; 95% CI: 24.4, 393.0; Cohen’s d = 0.61; *p* = 0.02) increased in the treatment group versus control. C-reactive protein also rose in the Healthycell group during the trial (t = 2.568; mean difference = 1.41 mg/dL; SE = 0.4; 95% CI: 0.3, 2.5; Cohen’s d = 0.66; p < 0.01), without accompanying increases in IL-6 and TNF-α. Additionally, cortisol levels decreased in the Healthycell group (t = 0.575; mean difference = −0.31 ug/dL; SE=0.1; 95% CI: −0.6, −0.03; Cohen’s d = 0.88; *p* = 0.03). When groups were split by age (< 35 years vs. ≥ 35 years), 8-hydroxydeoxyguanosine, a marker of DNA damage, decreased in the older Healthycell group compared to placebo (t = 1.782; mean difference = −7.09 ng/mL; SE = 3.0; 95% CI: −13.3, −0.9; Cohen’s d = 0.63; *p* = 0.03). Significant changes were also found for sTNFR-I, sTNFR-II, and IL-5 in the older group. All results were obtained from t tests by post-hoc analysis.

**Conclusions:** Our findings show an improved inflammatory profile and decreased DNA damage. Additionally, the efficacy of Healthycell was primarily in older adults, where the processes that cause or are associated with cell senescence are more predominant.

**Relevance for patients:** Healthycell may help to counteract the inflammatory effects of aging that lead to both cell senescence and the multitude of age-related chronic diseases.

## 1. Introduction

Chronic diseases, such as coronary artery and cardiovascular diseases, diabetes, cancer, and obesity [1-4], are widespread among the American population. In addition, the risk of developing multiple chronic diseases increases with age [5]. These diseases incur enormous public health costs [6,7], and poor nutrition is a key factor in their development [8-11]. Therefore, enhancing nutritional status is a well-founded strategy for addressing these concerns.

Why older persons are more susceptible to certain diseases is a complicated issue, though a convincing amount of evidence points to a declining immune system, i.e., immunosenescence [12,13]. The phenomenon of immunosenescence, which in part involves the reduced generation of T and B cells [14], leaves older individuals less able to defend themselves against illness. Along with a weakening immune system, the amount of adipose tissue in the abdominal region simultaneously increases with age [15]. This is especially concerning considering abdominal adipocytes secrete greater amounts of pro-inflammatory cytokines, such as TNF-α and IL-6, than their subcutaneous counterparts. Because these inflammatory mediators are linked to a variety of chronic diseases [15], older individuals may stand to benefit more from treatments that combat visceral fat. Another theory holds that aggregated pools of senescent cells may be culpable for the increased levels of pro-inflammatory mediators in older individuals [16]. Normally, senescent cells are recycled by the immune system, but when a sufficient volume of these cells aggregates, they may take on a senescence-associated secretory phenotype (SASP) in which detrimental pro-inflammatory molecules are released, potentially leading to a variety of age-associated diseases [16]. Because these rogue cells accumulate across the lifespan, presumably targeting these cells and their secreted inflammatory mediators earlier in life may represent a novel way to eliminate or reduce the risk of age-associated chronic disease. Thus, counteracting immunosenescence and its causes may be one method to impede the onset of age-related diseases.

Combating these aging and chronic disease issues through non-pharmacological modalities, such as dietary supplements, is an appealing option. However, improving host nutritional status with single-ingredient dietary supplements has largely failed. For example, a meta-analysis on the effects of vitamin and antioxidant supplements, the majority of which were administered as single ingredients, found little to no beneficial effects on cardiovascular disease [17]. Additionally, multi-nutrient supplements are not as commonly tested in clinical trials. In an *in vivo* study comparing omega-3 fatty acid supplementation to fish consumption, fish consumption produced beneficial effects on cerebrovascular disease, while omega-3 supplementation did not [18]. Presumably, the wider array of nutrients in the fish, such as its vitamins and trace elements, produced its superior results. We previously showed improvements in cognitive and immune functioning and inflammation in persons with Alzheimer’s disease in response to a dietary supplement containing multiple nutrients, such as polysaccharides, antioxidants, and omega-3 fatty acids, among others [19]. We also found that the combination of gingko biloba and choline produced modest improvements in cognitive functioning and inflammation in a sample of healthy elderly adults [20]. Thus, the continued investigation of multi-component formulations is warranted inasmuch as these studies provide the opportunity to evaluate a model of synergism among a complex of vitamins, minerals, phytonutrients, and co-factors. Why multi-nutrient supplements may offer better efficacy over single-ingredient supplements needs continued evaluation and delineation.

The purpose of this study is to evaluate the broad-spectrum dietary supplement Healthycell on key biomarkers of inflammation, oxidative stress, and DNA damage known to be involved in cellular aging in both somatic and stem cells. This product includes a wide range of nutrients and phytochemicals that are known to have anti-inflammatory effects, including resveratrol complex [21], L-arginine and L-citrulline [22], co-enzyme Q10 [23], phenolic acids [24-26], flavonoids [27], carotenoids [28], and several vitamins and minerals [29-32].

Two earlier versions of the product were compared with each other in a randomized trial over four weeks with reassessment after a two-week washout period [33]. Those data showed significant reductions in 8-hydroxydeoxyguanosine (8-OHdG), DNA adducts, and IL-lβ and a significant increase in plasma thiols, which are measures of DNA damage, inflammation, and DNA repair capacity, respectively. The current study seeks to replicate those findings and extend the understanding of the effects of these nutrients on objective data in an otherwise healthy population. Additionally, the supplement is expected to have a greater effect on the outcome measures of the older study participants.

## 2. Methods

### 2.1. Subjects

The study was conducted with the approval of the University of Miami Institutional Review Board for human subjects research (registry name: Clinicaltrials.gov; registry number: NCT02032693; available at: https://clinicaltrials.gov/ct2/show/NCT02032693). Using a two-tailed independent samples t-test in G-Power software, and assuming α=0.05, power=0.70, effect size (Cohen’s d)=0.95, and a 15% attrition rate, the calculated sample size was n=34 (17 in each group). Potential subjects were initially identified from physician referrals, the Medical Wellness Center, and the Department of Psychiatry and Behavioral Sciences at the University of Miami Miller School of Medicine, where the data were collected. Recruitment began in December 2013 and ended in April 2014, after sufficient subjects were enrolled. Forty subjects were screened over the phone for inclusion and exclusion criteria. Inclusion criteria were: (a) between 18 and 55 years of age; (b) English speaking; (c) willing to provide blood, urine, and saliva samples; (d) willing to provide informed consent to participate in the study; and (e) able to stop taking current dietary supplements two weeks prior to enrollment in the study if they were currently on a regimen. Exclusion criteria were: (a) a body mass index > 40 m/kg^2^; (b) participation in another related study within 30 days prior to baseline assessment; (c) currently smoking cigarettes or stopped smoking less than 6 months ago; (d) gastrointestinal disorders that could lead to uncertain absorption of the study supplements, such as inflammatory bowel disease (e.g., ulcerative colitis or Crohn’s disease), colostomy, or eating disorder; (e) actively receiving chemotherapy or radiation treatment for cancer; (f) any clinically significant abnormalities on the basis of medical history, physical examination, and/or vital signs in the judgment of the investigator and/or sub-investigator that would prevent participation in this study; (g) a diagnosis of a terminal illness; (h) if female, pregnant, breastfeeding, or intending to become pregnant within the next month; (i) insulin-dependent diabetes and/or taking metformin; (j) a diagnosis of HIV; or (k) an uncontrolled thyroid condition.

**Figure 1.**
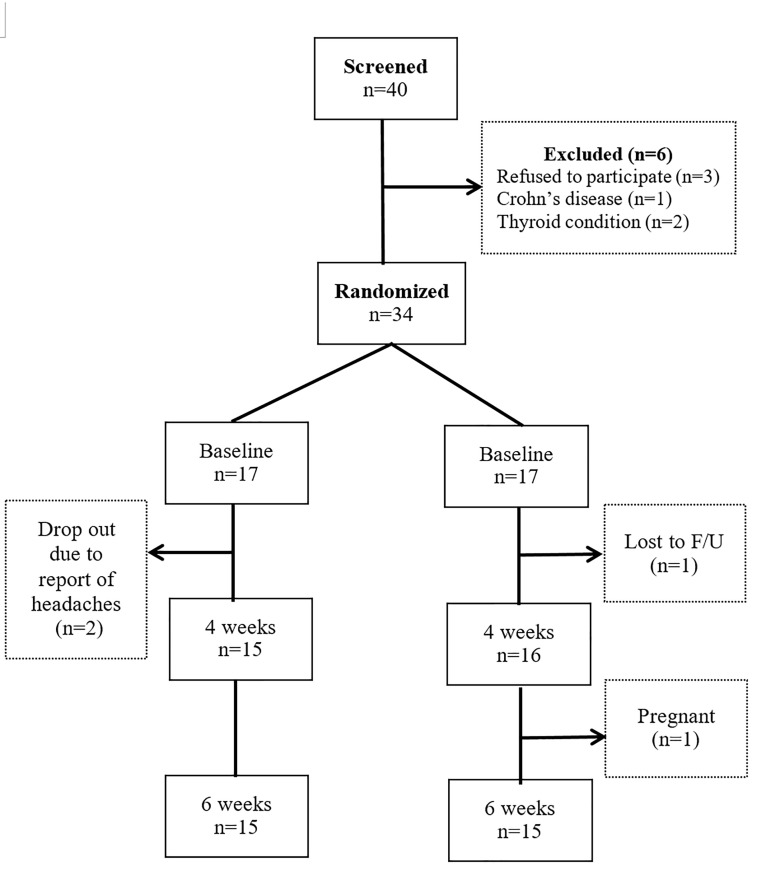
CONSORT flow diagram

Thirty-four subjects met the inclusion criteria and were enrolled in the study after signing the informed consent and HIPAA privacy forms prior to study entry. The participants were assigned using a simple randomization procedure to one of two conditions: (a) Healthy cell or (b) placebo, using a random permutations table, which balanced the number of participants in each group. All subjects and investigators were blinded to the treatment condition and remained blinded until after data analysis. Placebo and supplements were provided by CellHealth Institute (Montclair, NJ) labeled as Protocol A and Protocol B. Placebo consisted of colored tablets similar to those of the supplement and were made with cellulose. Only a staff member at CellHealth Institute knew the assignment of treatment to Protocol A or B. After randomization, participants were scheduled for assessments at baseline and weeks four and six, and blood was drawn at each time point to assess the biological markers. Each subject was compensated $50 for completing the assessments at each time point. One participant was withdrawn due to pregnancy, another for lack of compliance, and two subjects voluntarily withdrew after reporting headaches upon enrollment (See Figure 1). Thus, 30 participants completed the study.

### 2.2. Intervention

For the four-week intervention period, participants took either the Healthycell nutritional supplement or placebo (See Appendix 1 for the Healthycell product ingredients). Subjects took one tablet with eight ounces of water in the morning and evening, totaling two tablets per day. The four-week intervention period was followed by a two-week washout period. Subjects were advised not to modify eating or physical activity habits or prescription medication use during the study, unless directed by their physician. Subjects were instructed not to consume any other similar nutritional supplement for two weeks prior to having their baseline assessments and until the conclusion of the intervention and two-week wash-out period.

### 2.3. Outcomes and assessments

All participants completed an extensive sociodemographics and medical history questionnaire and reported their list of medications at baseline. They were also required to note any changes in type or amount of medication during the course of the study. The following biomarkers were assessed at each time point: (a) Type 1 T helper (TH1) pro-inflammatory cytokines − interferon gamma (IFNγ), tumor necrosis factor alpha (TNFα), tumor necrosis factor beta (TNFβ), interleukin-1 alpha (IL-lα), IL-1 beta (IL-1β), IL-2, IL-6, IL-8, IL-12, and IL-15; (b) Type 2 T helper (TH2) anti-inflammatory cytokines − IL-4, IL-5, IL-10, IL-13, IL-17, and IL-23; (c) soluble TNF receptor-I (sTNFR-I) and soluble TNF receptor-II (sTNFR-II) as inhibitors of the pro-inflammatory cytokine TNFα; (d) 8-hydroxydeoxyguanosine (8-OHdG) to measure DNA damage rate; (e) isoprostane as a marker of lipid peroxidation; (f) homocysteine as a methylation marker for gene expression; (g) c-reactive protein (CRP) as a general inflammatory marker; and (h) salivary cortisol as a marker of stress.

### 2.4. Biomarker assays

Blood samples were collected into ethylene diamine tetra acetic acid (EDTA) anticoagulant tubes. Plasma was separated within 2 hours of collection and stored at −80°C until assayed. The cytokines were measured in plasma using Quansys reagents and ELISA kit (Quansys Biosciences, Logan, UT) in the same way as reported previously in a larger cohort of chronic fatigue syndrome subjects with unknown etiology and illness trigger [34]. The range of the cytokine concentrations used in the standard calibration samples were adjusted for each cytokine along with sample exposure time. CRP was measured by a high sensitivity immunoelectro-chemiluminescence assay on a Roche Cobas 6000 analyzer (Roche Diagnostics, Indianapolis, IN) following manufacturer’s instructions. Inter-and intra-assay coefficients of variability (CV) for CRP were 2.5% and 4.2%, respectively. Urinary isoprostane levels were measured using an ELISA kit and reagents provided by Oxford Biomedical Research (Oxford Biomedical Research, Oxford, MI). Homocysteine was measured by enzymatic assay and cortisol by immunoassay on a Roche Cobas 6000 analyzer following manufactures instructions for assay and instrument set up (Roche Diagnostics, Indianapolis, IN), and inter-and intra-assay CV were less than 4.2% for both measures. 8-OHdG was measured by a competitive ELISA method using reagents provided by Cell Biolabs (San Diego, CA) with inter- and intra-assay CV of 3.8% and 4.9%.

### 2.5. Statistical analysis

Data were analyzed using SPSS 22 for Windows (IBM Inc., Chicago, IL). Frequency and descriptive statistics were calculated for all variables. The post-hoc test in the repeated measures analysis of variance (ANOVA) was used to assess changes over the course of the intervention between groups among the primary outcome variables. Post-hoc assessments were solely reported for several reasons. First, these comparisons alone were useful to interpret. The broader ANOVA results point only to the presence of an effect, and not where it is located. This is analogous to reporting multiple ANOVA results without including multivariate outcomes. Secondly, post-hoc tests are not dependent on significant ANOVAs, with the exception of the LSD post-hoc test, which was not used in this study [35]. Post-hoc tests were deemed significant at *p*< 0.05, Bonferroni corrections were used to correct for multiple comparisons, and results were obtained by conducting twotailed post-hoc assessments. In addition to comparisons conducted by treatment groups, analyses were conducted by age within treatment groups, i.e., younger group (< 35 years of age) vs. older group (≥35 years of age). If significant results were found, an independent or dependent t-test was performed (depending on the nature of the result) to obtain the necessary values to calculate the effect size (Cohen’s d).

**Table 1.** Sociodemographic characteristics of the sample

Variable	Category	Total Sample (n = 30)	Healthycell (n = 15)	Placebo (n = 15)	Statistic
Age	−	M = 36.9(SD =8.9;	M = 39.9(SD =9.8;	M = 33.9 (SD = 6.9;	t = –1.94, *p* = 0.06
		R = 21, 56)	R = 25.0, 56.0)	R = 21.0, 44.0)	
Gender	Male	8 (26.7%)	3 (20.0%)	5 (33.3%)	
	Female	22 (73.3%)	12 (80.0%)	10 (66.7%)	
Race/Ethnicity	White, non-Hispanic	4 (13.3%)	−	4 (26.7%)	
	Black, non-Hispanic	2 (10.0%)	1 (6.7%)	2 (13.3%)	
	Hispanic	15 (76.6%)	14 (93.3%)	9 (60.0%)	
Education	Up to High School	1 (3.3%)	−	1 (6.7%)	
	Post High School Training	2 (6.7%)	1 (6.7%)	1 (6.7%)	
	Some College	11 (36.7%)	6 (40.0%)	5 (33.3%)	
	College Graduate	4 (13.3%)	2 (13.3%)	2 (13.3%)	
	Master’s Degree	9 (30.0%)	5 (33.3%)	4 (26.7%)	
	Doctorate Degree	3 (10.0%)	1 (6.7%)	2 (13.3%)	
Marital status	Never Married	8 (26.7%)	3 (20.0%)	5 (33.3%)	
	Married	19 (63.3%)	10 (66.7%)	9 (60.0%)	
	Widowed	1 (3.3%)	1 (6.7%)	−	
	Divorced	1 (3.3%)	1 (6.7%)	−	
	Separated	1 (3.3%)	−	1(6.7%)	

Legend: M = mean, R = Range

## 3. Results

### 3.1. Safety and tolerability

During the entire study period, two subjects were withdrawn right after enrollment due to headaches of an unconfirmed nature that were not necessarily related to the study. In addition, four participants on the supplement reported sleepiness related to melatonin, and four subjects reported having a cold during the intervention. Otherwise, no adverse event was reported.

### 3.2. Sociodemographics and medication use

See Table 1 for the descriptive information of the sample for age, gender, race/ethnicity, education, and marital status, which were all non-significant between the Healthycell and placebo groups. The Healthycell group (n = 15) had 7 (47%) participants < 35 years of age and 8 (53%) participants ≥ 35 years of age. The placebo group (n = 15) had 8 (53%) participants < 35 years of age and 7 (47%) participants ≥ 35 years of age. Pre-specified analyses included examining groups without age splitting. However, exploratory analyses using post-hoc tests were done on age-split groups, and they revealed significant differences along these age differences. Bonferroni corrections were included to correct for type 1 error in all analyses.

At least one participant reported using a benzodiazepine, appetite suppressant, SNRI, prescription-grade multivitamin, decongestant, or antihistamine (each participant represented 3.3% of the cohort). Antacids, thyroid replacement hormones, and birth control were each separately reported in 6.7% of the subjects (n = 2). The most frequently used drugs, grouped into one category, were the pain relievers Tylenol, Advil, Motrin and ibuprofen, for which 19.4% of participants reported taking (n = 6). The two participants taking thyroid medications had euthyroidism due to their medication use, which would not inhibit the metabolism of the supplement, and therefore they were not excluded from the study. No significant differences were found between prescription and over-the-counter drug use between Healthycell and placebo groups.

### 3.3. TH1 pro-inflammatory cytokines

**Figure 2.**
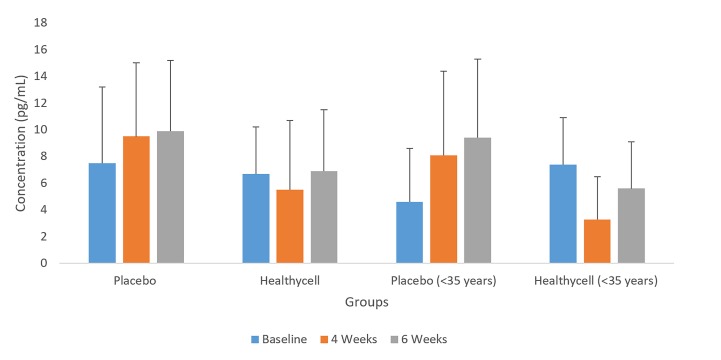
IL-1α in all-ages and younger Healthycell and placebo groups

IL-1α was not statistically different between the two groups at baseline (p = 0.52), but was significantly lower in the Healthycell group compared to the placebo group at the four-week assessment (t = 2.03; mean difference = −3.97 pg/ml; SE = 2.0; 95% CI: −8.0, α0.3; Cohen’s d = 0.77; p = 0.05). In addition, IL-lα increased from baseline to six weeks in the placebo’s younger group (t = 3.81; mean difference = 4.83 pg/ml; SE = 1.8; 95% CI: 0.1, 9.5; Cohen’s d = 1.13; p = 0.04; see Figure 2).

### 3.4. TH2 anti-inflammatory cytokines

**Figure 3.**
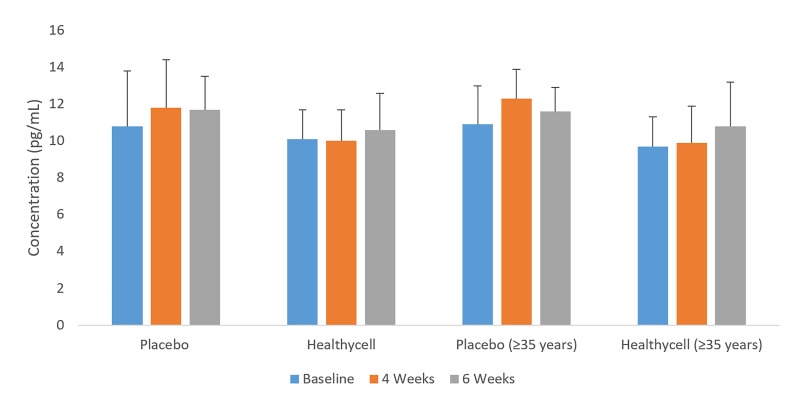
IL-2 in all-ages and older Healthycell and placebo groups

In the Healthycell group, IL-5 was higher in the older participants compared to the younger participants (t = 2.62; mean difference = 2.27 pg/ml; SE = 0.7; 95% CI: 0.9, 3.7; Cohen’s d = 1.46; *p* < 0.01) at four weeks, after being non-significant at baseline (*p* = 0.44). In older participants, IL-5 was higher in the Healthycell group compared to placebo (t = 2.20; mean difference = 1.51; SE = 0.7; 95% CI: 0.1, 2.9; Cohen’s d = 1.54; p = 0.04) at four weeks, following a non-significant baseline difference (p = 0.19). Additionally, in the Healthycell group, IL-5 increased (t = 2.57; mean difference = 1.73 pg/ml; SE = 0.6; 95% CI: 0.1, 3.3; Cohen’s d = 0.97;*p* = 0.03) from four to six weeks in the younger participants, while no changes were found in the older group across the same interval (*p* = 0.26). Among younger participants, IL-5 was lower in the Healthycell group (t = 2.89; mean difference = −.83 pg/mL; SE = 0.7; 95% CI: 0.4, 3.2; Cohen’s d = 1.60;*p* = 0.01) compared to the placebo group at the four-week assessment, while the difference at baseline was non-significant (*p* = 0.28; see Figure 3).

### 3.5. Soluble cytokine receptors

sTNFR-I increased from baseline to six weeks in the Healthycell group (t = 2.06; mean difference = 52.39 pg/mL; SE = 18.5 95% CI: 5.2, 99.6; Cohen’s d=0.53; *p* = 0.03). Additionally, sTNFR-I increased from baseline to four weeks (t = 1.90; mean difference = 46.52 pg/mL; SE = 17.9; 95% CI: 0.7, 92.4; Cohen’s d = 0.67; *p* = = 0.05; *p* = 0.05), four to six weeks (t = 2.08; mean difference = 57.82 pg/ml; SE=16.8; 95% CI: 14.8, 100.8; Cohen’s d = 0.74; *p* = 0.01), and baseline to six weeks (t=3.19; mean difference = 104.34 pg/ml; SE = 21.7; 95% CI: 48.8, 159.9; Cohen’s d = 1.13; *p* < 0.001), for the older participants in the Healthycell group. Within the Healthycell group, sTNFR-I was higher in older participants compared to younger participants at the six-week assessment (t = 1.90; mean difference = 161.09 pg/mL; SE = 72.0; 95% CI: 13.1, 309.1; Cohen’s d = 1.06; *p* = 0.04), but not at the baseline (*p* = 0.41) or four-week time points (*p* = 0.16; see Table 2).

sTNFR-II increased from baseline to six weeks (t = 1.74; mean difference = 208.71 pg/mL; SE = 72.0; 95% CI: 24.4, 393.0; Cohen’s d = 0.61; *p* = 0.02) among older participants in the Healthycell group. Within the Healthycell group, sTNFR-II was higher in older participants compared to younger participants (t = 1.75; mean difference = 246.6 pg/mL; SE = 109.2; 95% CI: 22.0, 471.1; Cohen’s d = 0.97; *p* = 0.03) at the six-week assessment (see Table 2).

### 3.6. Other biomarkers

**Figure 4.**
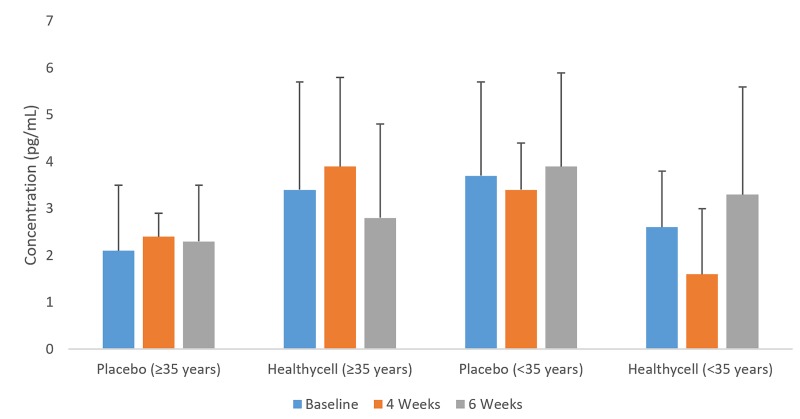
IL-5 in younger and older Healthycell and placebo groups

**Figure 5.**
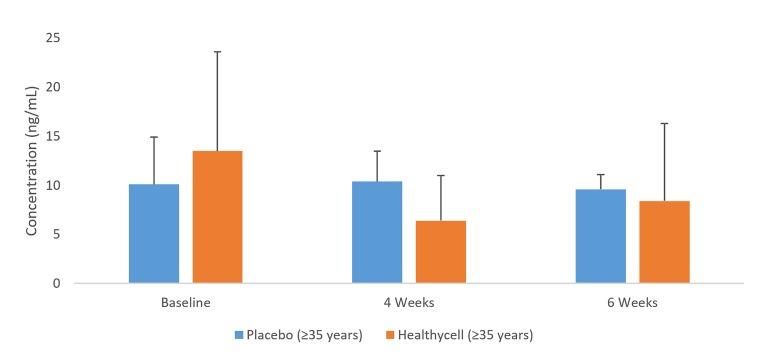
8-OHdG in older Healthycell and placebo groups

**Figure 6.**
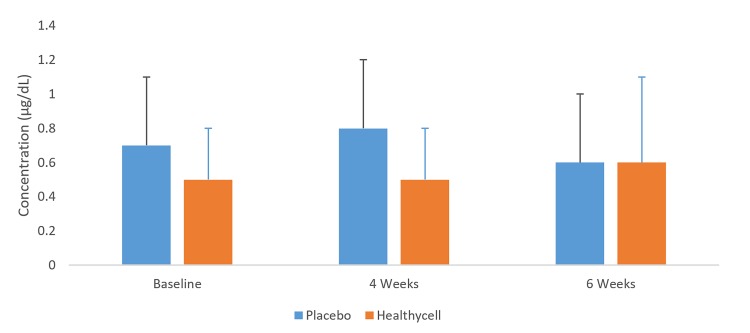
Cortisol in all-ages Healthycell and placebo groups

In the Healthycell group, 8-OHdG decreased among older participants from baseline to four weeks (t = 1.78; mean difference = −7.09 ng/ml; SE=3.0; 95% CI: −13.3, −0.9; Cohen’s d=0.63; p=0.03; see Figure 4). Salivary cortisol decreased in the Healthycell group compared to placebo at four weeks (t=2.28; mean difference = −0.31 ug/dL; SE = 0.1; 95% CI: −0.6, −0.03; Cohen’s d = 0.88; *p* = 0.03) after a non-significant difference at baseline (*p* = 0.11; see Figure 5). CRP increased in the Healthycell group from baseline to four weeks (t=2.57; mean difference = 1.41 mg/dL; SE = 0.4; 95% CI: 0.3, 2.5; Cohen’s d = 0.66; *p* < 0.01), although significance was lost when the sick participants (n=4) were excluded from the analysis (t=1.82; mean difference = 1.10 mg/dL; SE = 0.5; 95% CI: −0.7, 2.3; p = 0.07; see Figure 6). Lastly, neither homocysteine nor isoprostane levels changed throughout the intervention across or within groups.

**Table 2.** Soluble cytokine receptors with anti-inflammatory activities

Variable	Baseline	four weeks	six weeks
sTNFR-I placebo	354.5 ± 105.1	351.8 ± 103.2	358.9 ± 106.3
sTNFR-I placebo (< 35 years)	364.8 ± 91.2	359.7 ± 109.6	369.8 ± 97.7
sTNFR-I placebo (≥ 35 years)	342.8 ± 125.6	342.8 ± 103.3	346.5 ± 122.0
sTNFR-I Healthycell	335.9 ± 120.2^‡^	357.1 ± 167.8	388.3 ± 178.1^‡^
sTNFR-I Healthycell (< 35 years)	309.4 ± 65.2	301.5 ± 86.6	302.4 ± 71.1^†^
sTNFR-I Healthycell (≥ 35 years)	359.1 ± 154.8^*,***^	405.6 ± 210.0^*,**^	463.5 ± 212.8^**,***,†^
sTNFR-II placebo	765.9 ± 136.1	744.0 ± 137.6	723.9 ± 117.0
sTNFR-II placebo (< 35 years)	735.9 ± 138.0	729.9 ± 124.8	722.5 ± 125.4
sTNFR-II placebo (5= 35 years)	800.1 ± 135.8	760.2 ± 159.5	725.6 ± 116.6
sTNFR-II Healthycell	731.1 ± 149.5	769.0 ± 218.4	855.2 ± 291.9
sTNFR-II Healthycell (< 35 years)	696.3 ± 172.3	711.7 ± 209.3	723.7 ± 267.5^Ω^
sTNFR-II Healthycell (55 35 years)	761.5 ± 130.4^£^	819.1 ± 227.3	970.2 ± 277.0^£, Ω^

Legend: sTNFR-I and sTNFR-II are measured in pg/mL. Results were obtained in post-hoc tests following repeated measures ANOVA. All other comparisons were non-significant at a=0.05. sTNFR-I=soluble tumor necrosis factor receptor 1 and sTNFR-II=soluble tumor necrosis factor receptor 2. Mean ± SD (all such values). Footnotes: ^‡^sTNFR-I increased from baseline to six weeks (t=2.06; mean difference=52.39 pg/ml; SE=18.5 95% CI: 5.2, 99.6; Cohen’s d=0.53; p=0.03); *sTNFR-I increased from baseline to four weeks (t=1.90; mean difference=46.52 pg/ml; SE=17.9; 95% CI: 0.7, 92.4; Cohen’s d=0.67; p=0.05); “sTNFR-I increased from four weeks to six weeks (t=2.08; mean difference=57.82 pg/ml; SE=16.8; 95% CI: 14.8, 100.8; Cohen’s d=0.74; p=0.01); ^‡^sTNFR-I increased from baseline to six weeks (t=3.19; mean difference=104.34 pg/ml; SE=21.7; 95% CI: 48.8, 159.9; Cohen’s d=1.13; p<0.001); ^†^sTNFR-I was higher in older participants compared to younger participants (t=1.90; mean difference=161.09 pg/ml; SE=72.0; 95% CI: 13.1, 309.1; Cohen’s d=1.06; p=0.04); sTNFR-II increased from baseline to six weeks (t=1.74; mean difference=208.71 pg/ml; SE=72.0; 95% CI: 24.4, 393.0; Cohen’s d=0.61; p=0.02); ^Ω ^sTNFR-II was higher in older participants compared to younger participants (t=1.75; mean difference=246.6 pg/ml; SE=109.2; 95% CI: 22.0, 471.1; Cohen’s d=0.97; p=0.03).

**Figure 7.**
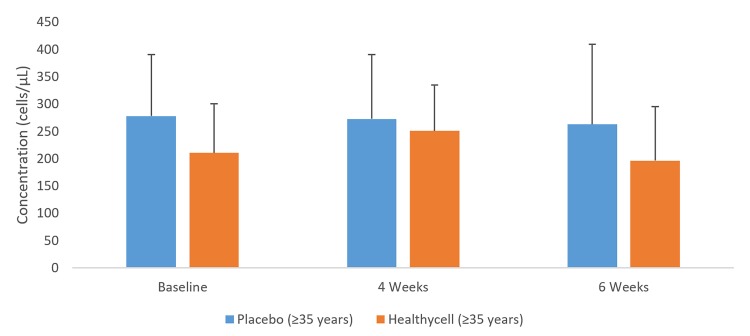
CD19+ Cells in older Healthycell and placebo groups

## 4. Discussion

The purpose of this study was to determine the four-week effects of the broad-spectrum dietary supplement, Healthycell, on various inflammatory, oxidative stress, and DNA damage biomarkers in healthy adults. Studies indicate that the primary drivers of cellular aging in both somatic and stem cell pools are DNA damage, free radical levels, telomere shortening, inflammation, and changes in gene expression, all of which slowly increase with advancing age [36,37].

We noted several consistent findings in the Healthycell group, such as the pro-inflammatory cytokine IL-lα decreased, while the anti-inflammatory cytokine IL-5 and soluble receptors sTNFR-I and sTNFR-II increased. Additionally, 8-OHdG, a marker of DNA damage, and salivary cortisol decreased, while CRP slightly increased, but without definite causation. Many improvements were observed in the older participants (∆ 35 years of age), thus indicating that persons older than 35 years of age may respond better to this product.

Flavonoids, a class of phenolic compounds found in Healthycell’s ingredients, have been found to have a wide variety of anti-inflammatory effects [24-26] and may partially account for our findings. For example, an *ex vivo* study found that the flavonoids fisetin and tricetin greatly attenuated LPS-induced increases in concentrations of TNF-α in COPD patient blood and IL-6 in type 2 diabetes patient blood by targeting poly (ADP-ribose) polymerase, an essential element in upregulating pro-inflammatory cytokines [27]. Another class of molecules with broad anti-inflammatory effects in the Healthycell dietary supplement are carotenoids [25,30,38,39]. Similar to phenolic compounds, carotenoids indirectly attenuate pro-inflammatory cytokines by their direct action on NF-κβ, an activator of many pro-inflammatory genes including those encoding inducible nitric oxide synthase, cyclooxygenase-2, TNF-α, IL-1β, and IL-6 [28]. Other ingredients known to have anti-inflammatory effects in this product include vitamins A, C, E, K, N-acetyl- L-cysteine, minerals such as zinc and selenium, and other cofactors such as coenzyme Q10 [29-32]. Thus, the positive changes we found in several pro- and anti-inflammatory cytokines would support the efficacy of Healthycell.

Although we did not find a specific effect of Healthycell on TNF-α, a pro-inflammatory cytokine known to be related to many diseases including rheumatoid arthritis, inflammatory bowel disease, psoriasis, psoriatic arthritis, and ankylosing spondylitis [40-42], we noted positive effects on sTNFR-I/-II. This is significant, given sTNFR-I/-IFs roles in inhibiting TNF-α in circulation; thus preventing TNF-α from interacting with membrane-bound receptors [42-44].

Small sample size may account for the increase in IL-lα in placebo’s younger group from baseline to six weeks. In addition, an interesting result was found for IL-5, in which somewhat opposite trends were found depending on which age group was analyzed through the four-week time point (see Figure 3). Although the younger Healthycell group showed a significant decrease in IL-5 relative to control at four weeks (p = 0.01) following non-significant baseline differences, the increase in the washout period was significant (*p* = 0.03). Thus, larger cohorts will be necessary to delineate the effects of this product on IL-5 levels. Our positive finding on 8-OHdG, a well-known marker of oxidative damage on DNA, is meaningful for at least two reasons. First, deoxyguanosine is the most likely base to be affected by oxidative species, as it has the lowest ionization potential of all of the DNA bases [45,46]. Second, 8-OHdG may also be a marker of carcinogenesis, although no direct link has been made between damaged DNA and cancer. Nonetheless, continual oxidative damage that results in the production of DNA lesions could lead to a lack of base pairing specificity and misreading of modified DNA bases, which are hallmarks of cancer development [46].

Our cortisol test results at first seem counter to the supposed anti-inflammatory effects of Healthycell because cortisol, like other glucocorticoids, typically acts in an anti-inflammatory manner. Thus, decreasing levels of cortisol would indicate pro-inflammatory consequences. However, recent data suggest the story is more complex. One group found that psychological stress induced by environmental triggers increased airway inflammatory responses to irritants, allergens, and infections in persons with asthma and children who were subjected to chronic stress (i.e., cortisol releasing) were associated with reduced expression of glucocorticoid receptor mRNA [47]. Moreover, stress is often accompanied by sleep deprivation, which in turn causes an elevation in cortisol and concomitant increases in pro-inflammatory cytokines [48]. Healthycell contains melatonin, which may be at least partially responsible for the decrease that we noted in cortisol. The melatonin may have increased the hours slept by participants per day, which could have also contributed to the reports of increased sleepiness.

Although CRP slightly increased in the Healthycell group over the four-week intervention period, CRP is typically induced by IL-6 and TNF-α, and neither parameter increased during the trial [12]. Moreover, CRP acts as a surrogate marker for these two cytokines, without which CRP does not predict mortality in survival analysis [12]. Lastly, our finding of slightly increased CRP may have been skewed by a few sick participants (n = 4). When we reanalyzed CRP with these participants excluded, the significant increase disappeared.

In summary, this study suggests that Healthycell exhibits an anti-inflammatory effect, and that this effect is best exhibited in individuals ∆35 years of age. It is also possible that this supplement could be used in a prophylactic manner. Studies have shown that long-term use of anti-inflammatory pharmaceutical options like aspirin can reduce the risk of colorectal cancer [49], that a combination of physical activity and healthy diet can lower vascular inflammation and insulin resistance [50], and that a vegetarian diet can produce long-term benefits that reduce the risk of cardiovascular disease via antioxidant foods [51]. With these positive results and further investigation, the dietary supplement Healthycell may be an option for persons concerned with their well-being in later years.

## 5. Limitations

The Healthycell formulation has a wide variety of phytonutrients, vitamins, and minerals that makes it impossible to delineate which nutrients may be responsible for the effects shown in the study. Also, it is difficult to determine which ingredients are synergistic with one another. Moreover, analyses included exploratory, multiple comparisons of age-split subgroups, which were subject to Bonferroni corrections, but may have still yielded false positive results. The minimal power (0.70) of the study may also have inhibited our ability to detect further effects. A future study that focuses on epigenetic markers and specific genes involved in the cellular aging processes (e.g., MTOR, AKT, and P53) may help clarify which ingredients or group of ingredients acted synergistically in producing these results. Additionally, the small sample size, healthy status, young age (M = 36.9 years; see Table 1), and the relatively short study intervention (four weeks) limit the generalizability of the results. What happens in a larger sample of older participants for a longer period of time is unknown. Although the company reports no adverse events during the time the product has been on the market, larger and lengthier studies will need to be conducted to demonstrate its efficacy and safety. Such studies are warranted because diets rich in antioxidants suggest efficacy in combating the immune components in several non-communicable diseases, and thus may provide an alternative or complement to pharmaceutical options [30].

## 6. Conclusions

High-quality dietary supplements that counteract the effects of inflammation, oxidative stress, and DNA damage may target the underlying causes of cellular aging and in turn improve the nutritional and cellular status of the individual. It is estimated that 29% of the world’s population will be aged ∆60 years by the year 2025 [52]. Thus, without proper health promotion (e.g., dietary supplementation, nutrition, and exercise), age-related chronic diseases such as cardiovascular disease, cancer, pulmonary conditions, and neurodegenerative disorders will continue to rise. Healthycell may offer a tool to counteract the negative effects of inflammation and oxidative stress that lead to late-stage diseases; with pronounced effects in individuals aged 35 and older. Enhancements in combating these predictors of cell senescence as we age could also decrease healthcare costs for the older population in general, as quality of life and health span increase [30,53]. We used the Healthycell product in the current study and showed that it has short-term positive effects on inflammation, oxidative stress, and DNA damage in healthy adults. Next steps in the evaluation process of this product would be to replicate the current findings for a longer period of time, with older adults, and perhaps in adults who have known inflammatory conditions.
